# Supplementary figures and images for: Standardized Nursing Terminologies and Electronic Health Records: A Secondary Analysis of a Systematic Review
Source: Healthcare (Basel). 2025 Aug 9;13(16):1952. doi: 10.3390/healthcare13161952 (PMC12385341; doi:10.3390/healthcare13161952)

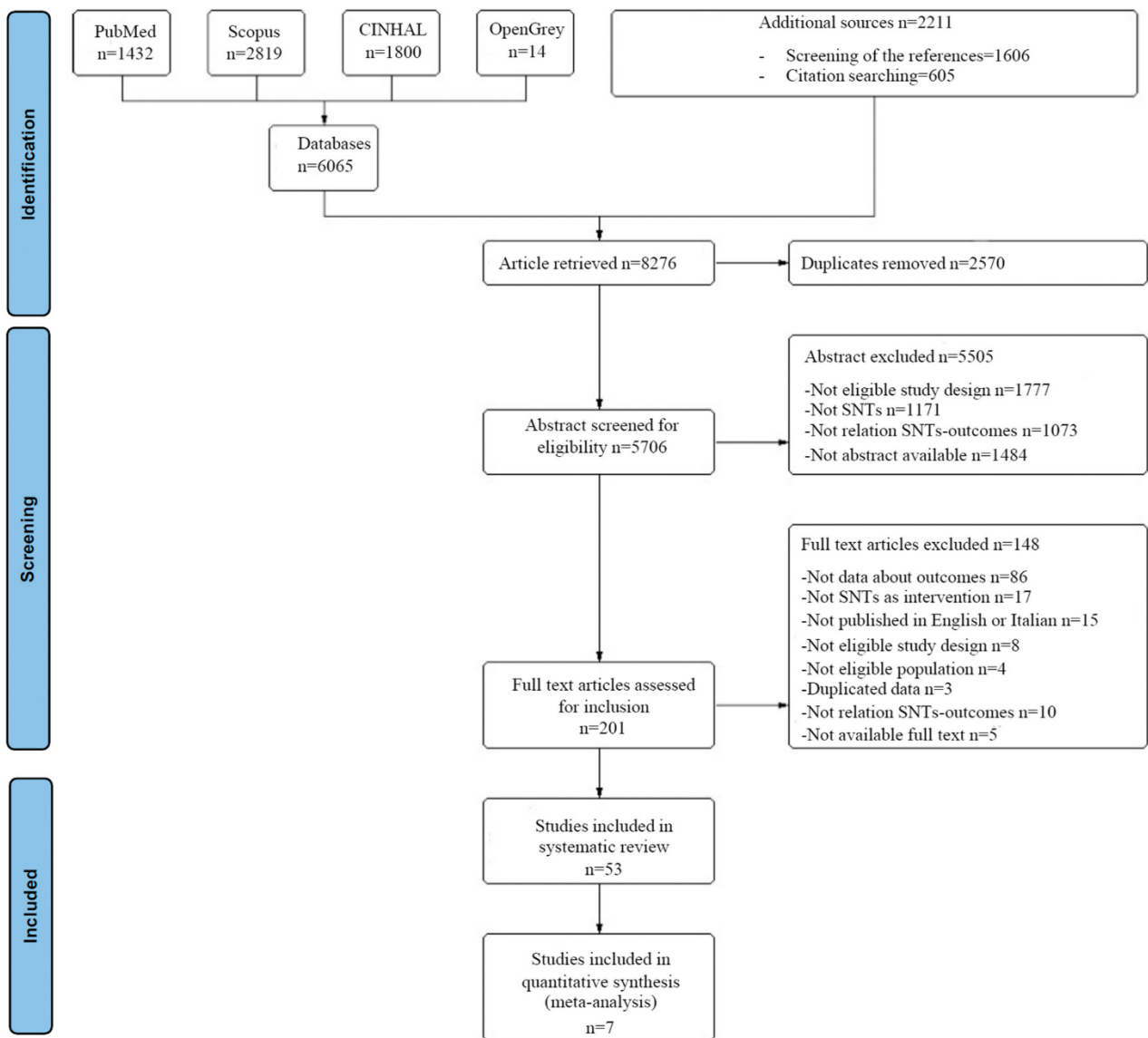

Figure S1. PRISMA flow diagram of included studies.

Supplement: Supplementary file 1 [file healthcare-13-01952-s001.zip › healthcare-3703055-supplementary.pdf]
